# Aortic wall abnormalities in patients with aortic coarctation

**DOI:** 10.1186/1532-429X-17-S1-P204

**Published:** 2015-02-03

**Authors:** Luis E Rodriguez Castellanos, Gabriela Melendez, Aloha Meave, Maria Soto, Jorge I Magaña

**Affiliations:** Magnetic Resonace, National Institute of Cardiology, Mexico City, Mexico

## Background

Aortic coarctation (AC) represents 7% of congenital heart disease. It is a reversible secondary cause of systemic hypertension, however up to 35% of patients remain hypertensive and 18% have cardiovascular complications such as aortic aneurysm, dissection or aortic valve disease. AC is associated with bicuspid aortic valve (BAV) in around 60%, both diseases alter aortic wall distensibility, stiffness and wall shear stress (WSS). Our objective was to compare these parameters in patients with AC according to the morphology or the aortic valve (either bicuspid or tricuspid).

## Methods

**Figure 1:**
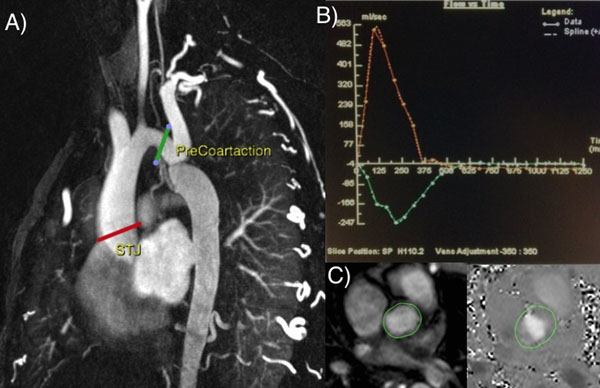
Magnetic resonance findings in patients with aortic coarctation and BAV.

Patients with AC or significant recoarctation (maximum pressure gradient >20 mmHg) who were referred for CMR from 2013 to 2014 were included. Patients with more than mild aortic valve disease and/or contraindications to undergo CMR were excluded. Distensibility was calculated at the sinotubular junction by the formula (systolic area - diastolic area)/((diastolic area x (systolic pressure - diastolic pressure)). We used pulse wave velocity (PWV), a validated subrogate of stiffness, which was measured as shown in figure 1.

## Results

Thirty-nine patients with AC were evaluated, nine were excluded due to moderate or severe aortic valve disease and one CMR was stopped due to claustrophobia; 29 patients were analyzed (17 with BAV and 12 with tricuspid aortic valve). There was no difference in distensibility between patients with BAV and tricuspid aortic valve [2.8 x 10^-3^ mmHg-^1^ (IQR 1.3-8.1) vs 3.8 x 10^-3^ mmHg-^1^ (1.7-5.3); p = 0.62], PWV was not different at precoarctation aorta [1.5 m/s (IQR 1.3-3.2) vs 2.2 m/s (1.1-4.6); p = 0.81] or at postcoarctation aorta [1.4 m/s (IQR 1.1-3.9) vs 3.3 m/s (1.2-10.8); p = 0.67]. Wall shear stress was similar in both groups (4.9 dynas/cm^2^ (IQR 4.1-6.0) vs 5.3 dynas/cm^2^ (2.9-8.3); p = 0.81]. Aortic diameters were not different between groups, however, we found a significant negative correlation between sinotubular junction diameter and WSS (r= -0.62; p=0.001) and distensibility (r=-0.47; p=0.02).

## Conclusions

Aortic distensibility, stiffness, WSS and diameters were similar in bicuspid and tricuspid aortic valve patients with aortic coarctation. Distensibility and WSS negatively correlate with the aortic diameter at the sinotubular junction.

